# Sexual Risk Behaviors of Male Current and Ex-opiate Users in Chiang Rai, Thailand

**DOI:** 10.2188/jea.12.345

**Published:** 2007-11-30

**Authors:** Pathom Sawanpanyalert, Saiyud Moolphate, Pornpimon Saksoong, Surachai Piyaworawong, Hideki Yanai

**Affiliations:** 1 TB/HIV Research Project, RIT-JATA, Chiang Rai Thailand; 2 Mae Chan Hospital, Chiang Rai Thailand

**Keywords:** HIV, opiate users, sexual behaviors, Thailand

## Abstract

Injecting drug use and unsafe sexual practice are both considered major risk factors for HIV infection. This study reports sexual behaviors among male “current” (i.e. using heroin and/or opium at least once in the past 3 months) and “ex-” opiate users in Chiang Rai province in Northern Thailand. Between January 1999 and August 2000, 206 male opiate users were recruited by mail callback. Of the 206 drug users, 89 (43.2%) could be classified as current users. Current users did not differ from ex-users, except for educational level and ethnicity. Current and ex-opiate users showed no difference in number of regular sexual partners, proportion of having sex with commercial and non-commercial sex partners, and reported histories of sexually transmitted diseases. This study suggests that the importance of sexual risk behaviors in HIV transmission cannot be ignored in both current and ex-opiate users.

Since early in the epidemic of human immunodeficiency virus (HIV)/acquired immunodeficiency syndrome (AIDS), drug injection and unsafe sexual behaviors have been recognized as major modes of transmission. It is currently estimated that there are about 800,000-1 million Thai people living with HIV/AIDS. Most of the infections are reported to be sexually transmitted, although transmission through drug injection is also a significant mode. However, injecting drug use has been limited to particular groups of population, and the proportion of HIV infections through injecting drug use is rather small, i.e. less than 10%. There are a few studies in Thailand that investigated potential, including sexual, routes of HIV transmission among drug users.^1^^,^^2^ However, due to much higher efficiency in transmitting HIV by drug injection, especially through sharing of drug paraphernalia, the role of sexual risk factors among drug users in HIV transmission is sometimes overlooked.^3^ In AIDS case surveillance, reported AIDS cases are usually assigned to only one risk category. Therefore, drug-using AIDS patients are rarely assigned to the sexual transmission risk group. This may result in underestimation of the importance of sexual risk behaviors in HIV infection among injecting drug users.

Evidence has shown that drug injectors are quite sexually active.^1^^,^^2^^,^^4^^-^^8^ They also tend to have multiple sex partners,^4^^,^^7^^-^^8^ practice unprotected sex,^3^^,^^7^ and use condoms inconsistently.^9^ Some drug injectors might also engage in activities that exchange sex for money or drugs.^8^^,^^10^ These factors can explain the spread of HIV infection from drug injectors to non-injectors, and from drug users to non-drug-using population. It is also suggested that persons who are at high risk of HIV infection through drug uses are more likely to be at risk of HIV through sexual behaviors than non-drug users.^11^ There is evidence showing that, despite significant reduction in drug use behaviors after substance abuse treatments, there have been very little change in their sexual behavior among drug users after the treatments.^8^^,^^12^^-^^16^ It is well recognized that prevention of HIV infection among drug users needs to go beyond providing use of new needles and syringes to cover unsafe sexual practices.^17^^-^^19^ Understanding sexual behaviors of injecting drug users is indispensable for any intervention programs aimed to reduce risk of HIV transmission among this group of people. This exploration is a part of a cohort study of opiate users in a hospital in Northern Thailand. It presents baseline data on sexual behaviors among male current and ex-opiate users. This information can be used to design appropriate interventions for the drug users.

## SUBJECTS AND METHODS

Chiang Rai is the northernmost province of Thailand. It has been hit hard by HIV/AIDS epidemic. More than 20,000 AIDS cases have been reported between 1988 and 2001 in Chiang Rai with about 25% already having died. The HIV serosurveillance among injecting drug users in Chiang Rai ranges from 20-60%. More than half of the injecting drug users share needles and/or syringes.

This study was conducted in a drug abuse treatment clinic in a community hospital in Chiang Rai. The community hospital with 90 beds belongs to the Ministry of Public Health of Thailand and treats the largest number of drug users in the province with approximately 60 to 100 new cases of injecting drug users per year in its substance abuse treatment clinic. The hospital is located about 30 km north of central Chiang Rai and treats about 50,000 outpatients per year. Methadone detoxification is the standard mode of treatment where a starting dose of 30-45 mg of methadone is used and later tapered to zero by the end of a 45-day treatment program. There has been no formal evaluation of this methadone detoxification program. Based on experiences of the physicians in charge of the clinic, however, a high rate of recidivism has been observed.

This study of opiate (heroin and/or opium) users was started in January 1999. The opiate users were recruited through mail callbacks. They were opiate users who previously completed the 45-day treatment program at the clinic within 10 years prior to the start of the study. Three letters, one month apart to the next letter, were sent to the opiate users in the clinic logbook to invite them to participate in a health study without specific mentioning of drug use, HIV/AIDS or sexual behaviors. No attempts were made to send more than 3 letters or make home visits as the investigators were afraid that it might induce panicking among the opiate users. Between the start of the study and August 2000, 1,049 opiate users were contacted and 214 (206 males and 8 females) showed up at the clinic as appointed and agreed to join the study. After signing a consent form, the opiate users were interviewed for demographics, drug use, sexual behaviors, and other risk behaviors. They were also tested for hepatitis C virus (HCV) antibody, HIV antibody, HBsAg and serologic test for syphilis (i.e. Venereal Disease Research Laboratory or VDRL). The participants were compensated for travel expenses at local rates. Since the number of female opiate users in this study was too small, it was decided later that they were not included in this report.

Interviews were done at the hospital’s clinic for drug users by two well-trained interviewers. The interviews were carried out in a private room with a comfortable and non-threatening environment. The interview usually lasted 20 to 30 minutes. The participants were informed of their rights to refuse any questions at any time without jeopardizing their relationships with the project staff and with the hospital. There were no participants who refused the interviews on sexual behaviors and other information.

With regard to sexual behaviors, the number of sexual partners, history of having sex with commercial and non-commercial sex workers, and history of sexually transmitted diseases were recorded. Sexually transmitted diseases among males include urethral discharge or pus, wart-like genital lesions, painful or painless genital ulcers, inguinal mass, genital blisters, and painful urination.

Insti HIV1/HIV2^®^ (Intracel Corporation, Cambridge Massachusetts) and TestPack HIV-1/HIV-2^®^ (Abbot GmbH, Delkenheim Germany) were used to detect antibody to HIV. Both of the tests were used interchangeably. No immunoblotting was done. The laboratory test for HBsAg was IM_X system HBsAg v2 (Abbot GmbH, Delkenheim Germany). Antibody to HCV was determined by using an enzyme immunoassay (EIA) HCV test kit, IM^®^X system HCV version 3.0 (Abbot GmbH, Delkenheim Germany) and confirmed by another EIA HCV test kit, Cobas^®^ Core Anti-HCV EIA (F. Hoffmann- La Roche, Basel Switzerland). VDRL (VDRL Carbon Antigen, Cambridge Massachusetts) tests were done for Treponema pallidum infection. The titer of 1:2 or more is considered reactive for VDRL testing.

Data were then coded and entered into a computer database and analyzed by Epi Info 6.04b and Stata release 6. Mean and standard deviation were used to describe continuous variables with approximate normal distribution. Median was used instead for data with wide dispersion. Proportion was used to describe categorical data.

“Current opiate users” were defined as those who used heroin and/or opium at least once in the past 3 months at the time of interview. Otherwise, they were classified as “ex-users”. Current opiate users were compared to ex-users. Differences in distribution of categorical independent variables were examined by chi square tests, except when the expected frequency was less than 5 and Fisher’s exact test was indicated. For continuous data, unpaired Student’s t tests were used for evaluating differences between two groups. Educational level was classified into three groups, i.e. no formal education, lower primary (grades 1-4), and upper primary (grades 5, 6, or 7) and higher. Odds ratios (OR) and their 95% confidence intervals (CIs) were calculated to determine the strength of association between the dependent variable (i.e. being current or ex-users) and various independent variables.

## RESULTS

There were 208 male opiate users agreeing to join the study. We observed no differences between opiate users who showed up and did not show up in terms of age, gender, and location of residence. Out of the 206 users, 89 (43.2%) were classified as current users. Characteristics of current and ex-users are shown in Table 1. Current and ex-users did not differ by age, marital status, occupation, yearly income, and drug injecting. Current users were more likely to have no formal education, and be of non-Thai ethnic group than ex-users. Although the prevalence of anti-HIV positivity, anti-HCV positivity, HBsAg positivity, and VDRL reactivity among current users were lower than those of ex-users, the differences did not reach statistical significance.

**Table 1.** Characteristics of male current and ex-opiate users in a drug abuse treatment clinic in Chiang Rai, Thailand.

Characteristic	Current (n=89)	Former (n=117)	OR (95% CI)	p value
Age (years)				
Range	20-67	20-74		
Mean	38.61	37.79		0.468
Standard deviation (SD)	12.24	13.10		
Median	37	37		

Education				
No formal training	35	21	Referent	
Lower primary	24	58	0.25 (0.12-0.51)	<0.001
Upper primary and higher	30	38	0.47 (0.23-0.98)	0.043

Ethnicity				
Thai	52	100	Referent	
Non-Thai	37	17	4.19 (2.15-8.14)	<0.001

Marital status				
Single	30	46	Referent	
Ever married	59	71	1.27 (0.72-2.26)	0.409

Occupation				
Unemployed	20	18	Referent	
Employed	69	99	0.63 (0.31-1.27)	0.309

Yearly income (Thai Baht*)				
Range	0-150,000	0-360,000		
Mean	32,759.89	44,145.13		0.173
Standard deviation (SD)	24,671.86	44,211.35		
Median	30,000	36,000		

Drug use behavior				
Exclusive non-injecting	36	47	Referent	
Ever injecting	53	70	0.99 (0.56-1.73)	0.968

% Anti-HIV positive	24/89	36/117	0.83 (0.45-1.53)	0.552
	(27%)	(31%)		

% Anti-HCV positive	51/88**	76/115**	0.71 (0.40-1.25)	0.236
	(58%)	(66%)		

% HBsAg positive	8/88**	16/116**	0.63 (0.25-1.53)	0.305
	(9%)	(14%)		

% VDRL reactive	1/89	4/116**	0.32 (0.03-2.90)	0.310
	(1%)	(3%)		

*Approximately 45 Thai Baht = 1 US dollar in December 2001

**The denominator is not exactly the same as the total because some cases have missing laboratory results.

As shown in Table 2, no differences were found between current and ex-users in terms of sex with regular sex partners, sex with female commercial and non-commercial sex partners, exchange of sex for money, and reported sexually transmitted diseases.

**Table 2.** Sex-related behaviors of male current and ex opiate users in a drug abuse treatment clinic in Chiang Rai, Thailand.

Characteristic	Current (n=89)*	Former (n=117)*	p value
Number of regular sex partners in the past 3 months			
Range	0-2	0-3	
Mean	0.58	0.66	0.774
Standard Deviation	0.52	0.62	
Median	1	1	

Having at least one regular sex partner in the past 3 months			
	29/79	40/100	0.653
	(37%)	(40%)	

Having sex with female non-commercial casual sex partners at least once in the past 3 months			
	0/88	1/114	0.564
	(0%)	(1%)	

Having sex with female commercial sex workers at least once in the past 3 months			
	1/88	1/114	0.683
	(1%)	(1%)	

Sex with female non-commercial casual sex partners in the past 12 months			
*Average monthly frequency (times per person)*			
Number of responding subjects	82	58	
Range	0-3	0-10	
Mean	0.07	0.24	0.229
Standard Deviation	0.37	1.40	
Median	0	0	
Regular condom use (100% use)	2/4	0/3	not calculated**
	(50%)	(0%)	

Sex with female commercial sex workers in the past 12 months			
*Average monthly frequency (times per person)*			
Number of responding subjects	82	58	
Range	0-3	0-1	
Mean	0.09	0.07	0.683
Standard Deviation	0.45	0.21	
Median	0	0	
Regular condom use (100% use)	5/6	6/7	not calculated**
	(83%)	(86 %)	

Reporting ever exchanging sex for money			
	0/88	0/112	not calculated**
	(0%)	(0%)	

Reporting any one of STD symptoms*** in the past 3 months			
	36/89	67/117	not calculated**
	(40%)	(57%)	

* The denominator for each variable may not be exactly equal to these totals because of missing data.

** Test statistics and p values were not calculated because of small number of responses.

*** Refer to the text for definition of reported STD symptoms.

## DISCUSSION

Among opiate users who participated in this study, current and ex-users are different in terms of educational level and ethnicity. However, they are quite similar in various other aspects that might have effects on sexual behaviors and/or HIV infection.^20^ Age is probably the strongest factor that determines sexual behaviors. In this study, however, both current and ex-users were comparable in terms of age. Therefore, it is unlikely that age accounts for differences or lack of differences between the current and ex-users. In addition, the two groups were not different in other important variables that might affect sexual behaviors. The sexual behaviors between the two groups, as measured by sex with regular partners and commercial and non-commercial casual sex partners, were not very different.

It might be possible that opiate users who showed up and joined the study differ significantly from those who did not show up. However, since investigators made no mentioning about studies of sexual behaviors or any HIV-related factors when they contacted the potential participants, it is unlikely that the differences would bias the results of this study. The external validity of this study should therefore still be intact. Nonetheless, reporting on sexual behaviors is a sensitive issue and is usually associated with under-reporting. Therefore, the levels of sexual activities in this study might be underreported. There are needs to use other methods to validate the sexual behavioral data. However, the degrees of underreporting between the two groups should be non-differential.

The slightly higher prevalence of positivity with anti-HIV antibody, anti-HCV antibody, HBsAg, and reactivity with VDRL among ex-users are not very surprising. It is possible that the ex-users might have been exposed to more cumulative risk of contracting infectious diseases, particularly in pre-harm reduction times, than the current users. This can explain the differences in the prevalence. However, the differences were not statistically significant.

Age range is quite wide. No attempts were made to divide the users into several groups of age, however, because the number in each group would be too small. Another major limitation of this study is that it did not include female opiate users in the analyses. Females are usually underrepresented in most studies related to drug use and HIV/AIDS. It is therefore necessary to encourage more studies on HIV/AIDS and drug use in females. If similar studies are to include female opiate users, they might have to put more emphases on issues of commercial sex. In addition, the definition of sexually transmitted diseases will need to be modified.

With the changing patterns of drug use in Thailand from opiates to amphetamine-type substance, it will be necessary to study sexual risk factors among users of amphetamine-type substance with or without opiates. In addition, it will be useful to see if amphetamine-type substance users are put to be at high risk of HIV transmission, either through their sexual behaviors or drug use behaviors.
